# No Consistent Evidence of Decreased Exposure to Varicella-Zoster Virus Among Older Adults in Countries with Universal Varicella Vaccination

**DOI:** 10.1093/infdis/jiab500

**Published:** 2021-10-05

**Authors:** Stephane Carryn, Brigitte Cheuvart, Michael Povey, Alemnew F Dagnew, Rafael Harpaz, Robbert van der Most, Giacomo Casabona

**Affiliations:** 1 GSK, Rixensart, Belgium; 2 GSK, Wavre, Belgium; 3 GSK, Rockville, Maryland, USA; 4 Harpaz-Herman Consultants LLC, Atlanta, Georgia, USA

**Keywords:** varicella vaccine, boosting, exposure, herpes zoster

## Abstract

**Background:**

Universal varicella vaccination might reduce opportunities for varicella-zoster virus (VZV) exposure and protective immunological boosting, thus increasing herpes zoster incidence in latently infected adults. We assessed humoral and cell-mediated immunity (CMI), as markers of VZV exposure, in adults aged ≥50 years.

**Methods:**

We repurposed data from placebo recipients in a large multinational clinical trial (ZOE-50). Countries were clustered based on their varicella vaccination program characteristics, as having high, moderate, or low VZV circulation. Anti-VZV antibody geometric mean concentrations, median frequencies of VZV-specific CD4 T cells, and percentages of individuals with increases in VZV-specific CD4 T-cell frequencies were compared across countries and clusters. Sensitivity analyses using a variable number of time points and different thresholds were performed for CMI data.

**Results:**

VZV-specific humoral immunity from 17 countries (12 high, 2 moderate, 3 low circulation) varied significantly between countries (*P* < .0001) but not by VZV circulation. No significant differences were identified in VZV-specific CMI between participants from 2 high versus 1 low circulation country. In 3/5 sensitivity analyses, increases in CMI were more frequent in high VZV circulation countries (.03 ≤ *P* < .05).

**Conclusions:**

We found no consistent evidence of reduced VZV exposure among older adults in countries with universal varicella vaccination.

**Clinical Trials Registration:**

NCT01165177.

## Teaser

By repurposing humoral and cell-mediated immunogenicity data from a large multinational trial enrolling adults aged ≥50 years, we found no consistent evidence of differences in varicella-zoster virus exposure between countries with widely circulating varicella versus countries with universal vaccination.

**(See the Editorial Commentary by Gershon and Gershon, on pages 361–3.)**

Varicella-zoster virus (VZV) is a human alphaherpesvirus that infects most individuals, usually during childhood, although its incidence may be delayed in warmer climates [1]. Primary infection with VZV produces varicella (chickenpox), which is usually a benign disease. VZV remains latent in neurons and may reactivate symptomatically later in life, causing herpes zoster (shingles). The risk of developing herpes zoster increases steeply with age once individuals reach 50 years; other risk factors for herpes zoster include sex, ethnicity, and immunosuppression [2, 3]. While the incidence of herpes zoster is lower than that of varicella, it is frequently associated with potentially debilitating complications such as postherpetic neuralgia and uveitis, generating a considerable aggregate health and economic burden [2, 4].

In 1965, Hope-Simpson hypothesized that VZV reactivation is under an immunological control that is periodically boosted by exposures to varicella (ie, exogenous boosting) and/or by prior subclinical VZV reactivation events (ie, endogenous boosting) [5]. The exact nature of the immunological mechanisms that mediate protection against herpes zoster continues to remain elusive. Therefore, with current knowledge, protective boosting cannot be demonstrated using any specific immunological measures. However, an increase in any VZV-related immunological outcome, irrespective of its protective effect against herpes zoster, provides proof that the immune apparatus has encountered VZV and has been stimulated (ie, has experienced a subclinical reinfection). Conversely, no increase of any immunological measure raises the possibility that the immune system has not been sufficiently exposed to VZV, meaning that exogenous boosting could not have occurred either. Thus, measurements of immunological outcomes can provide important information on the adequacy of VZV exposure [3].

Based on the observation that herpes zoster incidence was higher in adults living without children compared to those living with children (a surrogate for VZV exposure), Brisson et al [6] constructed a mathematical model that predicted an important increase in herpes zoster incidence following introduction of varicella vaccination due to a decrease in exogenous boosting. Even if the protective exogenous boosting phenomenon is valid, lower occurrence of varicella could cause important population-level increases in herpes zoster only if varicella exposures are measurably more frequent in varicella-endemic settings compared to those with well-controlled varicella. Stated differently, the portion of adults in the general population experiencing the intense and prolonged varicella exposures seen in affected households may be too small to measurably control herpes zoster, even in varicella-endemic settings [3].

Several countries have implemented universal varicella vaccination [7], leading to striking declines in varicella incidence, especially when 2 doses are administered [8–10]. However, concerns regarding the exogenous boosting hypothesis continue to elicit caution in policymakers contemplating introduction of universal varicella vaccination [3].

For this post hoc analysis, we repurposed data from a multinational randomized clinical herpes zoster vaccine trial [11] to determine whether universal varicella vaccination programs reduce the opportunities for VZV exposure and subsequent exogenous boosting, assessed by the prevalence of VZV-specific immunological outcomes as markers of VZV exposure. A summary contextualizing the outcomes of this analysis is displayed in the plain language summary (Box 1).

Box 1. **Plain Language Summary**

**What is the context?**

- The varicella-zoster virus, which causes varicella in childhood, remains latent and may reactivate later in life, leading to herpes zoster disease.
- Protection against varicella-zoster virus reactivation and herpes zoster is expected to be boosted by 2 mechanisms: exposure to varicella (exogenous boosting) and/or by asymptomatic reactivation of the virus (endogenous boosting). To induce exogenous boosting, the varicella exposure should be intense enough to stimulate an immune response.
- Varicella vaccines are highly efficacious in preventing varicella disease in children and are routinely used in several countries. Many other countries have delayed implementation based on the hypothetical concern that decreased exposure to varicella (through vaccination) may lead to an increase of herpes zoster disease in older adults, due to the lack of exogenous boosting.

**What is new?**

- We assessed the differences in varicella-zoster virus immune status among participants ≥ 50 years of age using samples from a clinical trail conducted in 18 countries. The objective was to seek evidence that there are more opportunities for intense varicella exposure in countries with endemic varicella (high virus circulation) compared to those with varicella vaccination programs (low virus circulation).
- We did not detect any differences in immunity among populations from different countries.

**What is the impact?**

- In our analysis, we found no consistent evidence that varicella vaccination programs reduce opportunities for varicella-zoster virus exposure among older adults.
- The results should be carefully considered by national authorities when implementing vaccination policies.

## METHODS

### Study Design and Participants

This post hoc exploratory analysis was based on data derived from placebo recipients in a large, placebo-controlled, randomized clinical trial of the adjuvanted recombinant zoster vaccine, conducted in 18 countries across North America, Latin America, Europe, Asia, and Australia, between August 2010 and July 2015 (ZOE-50, NCT01165177) [11]. Study participants included adults aged ≥50 years without history of herpes zoster or of immunization against varicella or herpes zoster. Detailed inclusion and exclusion criteria are described in the Supplementary Material. Participants were stratified by age group (50–59, 60–69, and ≥70 years) and randomized (1:1) to receive 2 doses of recombinant zoster vaccine versus NaCl placebo, 2 months apart. Blood for assessing humoral immune response was collected at months 0 and 3, and from a subset of these participants at months 14, 26, and 38. Cell-mediated immunity (CMI) was assessed in blood collected from a subset of the humoral immunity cohort at months 0, 3, 14, 26, and 38.

Cumulative effects of VZV exposure prior to the study, captured by the level of VZV-specific humoral and CMI responses at month 0, served as baseline, while potential changes in immunity caused by different rates of varicella exposure during the study were assessed at subsequent time points.

Written informed consent was obtained for all study participants prior to any study-specific procedure. ZOE-50 was conducted in compliance with the Declaration of Helsinki, the principles of good clinical practice, and applicable regulatory requirements. As only additional statistical analysis of preexisting data was performed for this study, reconsent was not needed.

### Clustering of Countries

**Figure 1.**
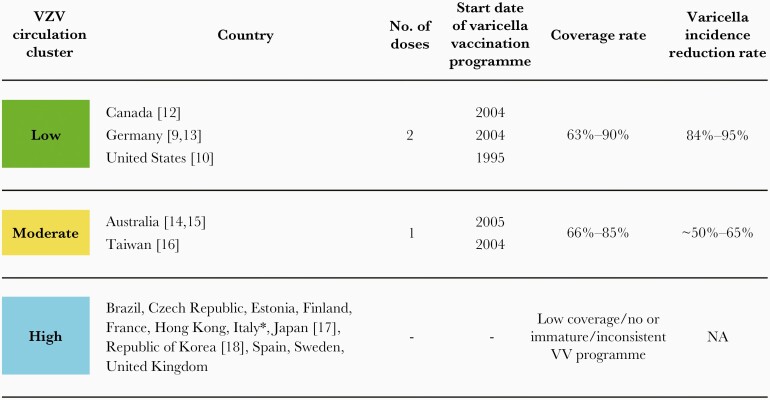
Grouping of countries according to their estimated VZV circulation level. Low, low VZV circulation: countries with a well-established 2-dose pediatric universal VV program. Moderate, moderate VZV circulation: countries with a well-established 1-dose pediatric universal VV program. High, high VZV circulation; countries where VV was not included in the national immunization program, the immunization program was immature or has suffered significant interruptions over time. ∗High coverage centers were excluded. Abbreviations: NA, not applicable; VV, varicella vaccination; VZV, varicella-zoster virus.

Countries were clustered according to the presence, length, and characteristics of their national pediatric varicella vaccination programs at the time of ZOE-50. Three categories were prespecified based on the presumed level of VZV circulation: low, moderate, and high (Figure 1) [9, 10, 12–18]. To support the validity of this clustering criteria, data regarding varicella incidence reduction achieved at the time of sample collection was also included.

### Anti–Varicella-Zoster Virus Antibody Testing

All humoral immunity tests were performed at the GSK Biologicals Global Vaccines Clinical Laboratories (Clinical Laboratory Sciences, Rixensart, Belgium). Serum anti-VZV antibody concentrations were evaluated using anti-VZV whole-virus–based enzyme-linked immunosorbent assay (anti-VZV enzyme-linked immunosorbent assay [ELISA], Enzygnost; DiaSorin, formerly Siemens). Anti-VZV ELISA has been validated to measure the immune response against live attenuated varicella vaccination [19]. The assay has a technical cutoff of 25 mIU/mL.

### Anti–Varicella-Zoster Virus Cell-Mediated Immunity Testing

All VZV-specific CMI responses were measured at the Center for Vaccinology, Ghent University, Belgium, on peripheral blood mononuclear cells by intracellular cytokine staining. Cryopreserved peripheral blood mononuclear cells were stimulated using VZV-lysate for 2 hours, after which brefeldin A was added. Following a further 18 hours incubation at 37°C, cells were stained with antibodies against CD4 and CD8 and permeabilized. Intracellular cytokine staining was performed using antibodies to interferon-γ (IFN-γ), tumor necrosis factor-α (TNF-α), CD40 ligand (CD40L), and interleukin 2 (IL-2). Details of the intracellular cytokine staining have been previously reported [20]. For the purpose of the analysis presented here, the frequency of CD4 T cells expressing at least IFN-γ and another activation marker (CD4^IFN-γ+(+)^ T cells) per 10^6^ CD4 T cells was assessed at each of the 5 time points, according to country. The focus on IFN-γ–secreting cells was based on the assumption that CD4 T cells induced by VZV viral infection would have a T helper 1-biased phenotype [21].

### Cell-Mediated Immunity Increase Analysis

A transient increase in CMI, defined as a VZV-specific CD4 T-cell frequency exceeding by at least twice the standard deviation (SD) of the individual predicted frequency at any time point, was used as a proxy for natural exposure to VZV at an individual level. The primary analysis was based on participants with measurements available for the first 4 time points.

### Statistical Analyses

All humoral and CMI analyses were post hoc analyses, based on the per-protocol cohorts for humoral and CMI. For each time point, the corresponding per-protocol cohort from ZOE-50 was used (adapted per-protocol cohort), including only participants in the placebo arm who met all eligibility criteria, complied with the protocol, and had immunity data available at each time point.

Geometric mean antibody concentrations (GMCs) were calculated by taking the antilog of the mean of the log concentration transformations. GMCs with 95% confidence intervals (CI) were further calculated by country and cluster of countries, based on the anti-VZV antibody concentrations detected at each time point. Comparisons between countries and clusters of countries were performed with a repeated linear mixed model to account for repeated measurements over visits. The model investigated country as fixed effect. To investigate VZV circulation, VZV was used as a fixed effect and country as a random effect. The estimates in each country or each VZV circulation cluster were back transformed to the original scale.

The frequency of VZV-specific CD4^IFN-γ+(+)^ T cells was calculated as the difference between the frequency of CD4^IFN-γ+(+)^ T cells stimulated in vitro with antigen versus culture medium alone. SD (calculated per participant) for CMI increase analysis was derived from a repeated measurement with country as fixed effect and participant as a random effect. An increase was defined as a VZV-specific CD4 T-cell frequency exceeding by at least 2 times the SD of the predicted frequency. The percentage of participants showing at least 1 increase was computed by country with 95% CI and was compared between countries using Fisher exact test. Due to the limited data included, and because no CMI correlate of protection has been established for herpes zoster, a sensitivity analysis was included. The same calculations were also performed on participants with CMI results for at least 2 time points (to include all data) and for all 5 time points (the most relevant results, but with a low number of participants), and with the CMI transient increase defined as a VZV-specific CD4 T-cell frequency exceeding by at least 3 times the SD of the predicted frequency (to explore whether increasing the signal-to-noise ratio results in a different effect).

## RESULTS

### Study Participants

At month 3, 1067 and 218 participants from the placebo arm of ZOE-50 were included in the per-protocol cohorts for humoral and CMI, respectively. Mean age of participants was similar in both cohorts: 64.6 years (SD 9.0) and 64.5 years (SD 8.9), respectively. The majority of participants were female (643 [60.3%] of 1067 and 119 [54.6%] of 218) and of Caucasian/European heritage (747 [70.0%] of 1067 and 128 [58.7%] of 218). Characteristics of the overall study population enrolled in the placebo arm of ZOE-50 have been previously published [11].

### Anti–Varicella-Zoster Virus Humoral Immune Status

Humoral immune samples were available from 17 countries: 12 with high, 2 with moderate, and 3 with low circulation. Humoral immune status at month 0, in terms of anti-VZV immunoglobulin G (IgG) GMC was similar in placebo and vaccine recipients in the ZOE-50 trial (1036.6 [95% CI, 981.9–1094.4] mIU/mL versus 945.5 [95% CI, 894.4–999.6] mIU/mL).

**Figure 2.**
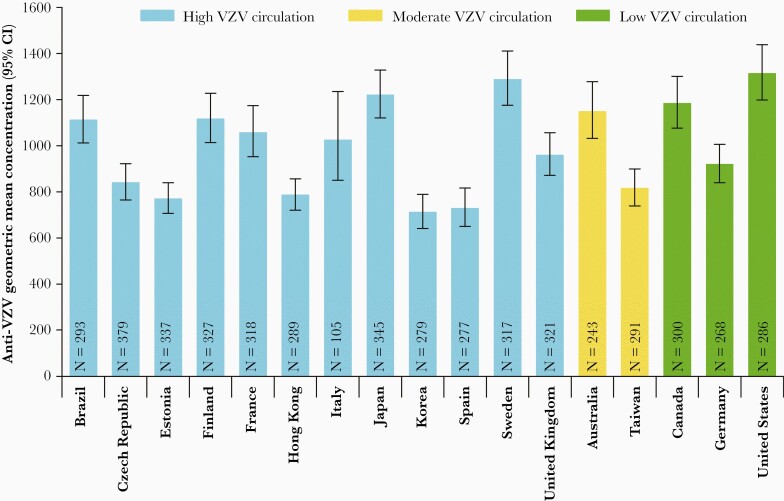
VZV-induced humoral responses across time by country (adapted per-protocol cohort for humoral immunity). Results from each participant were treated as a unit. Abbreviations: CI, confidence interval; N, number of participants; VZV, varicella-zoster virus.

**Figure 3.**
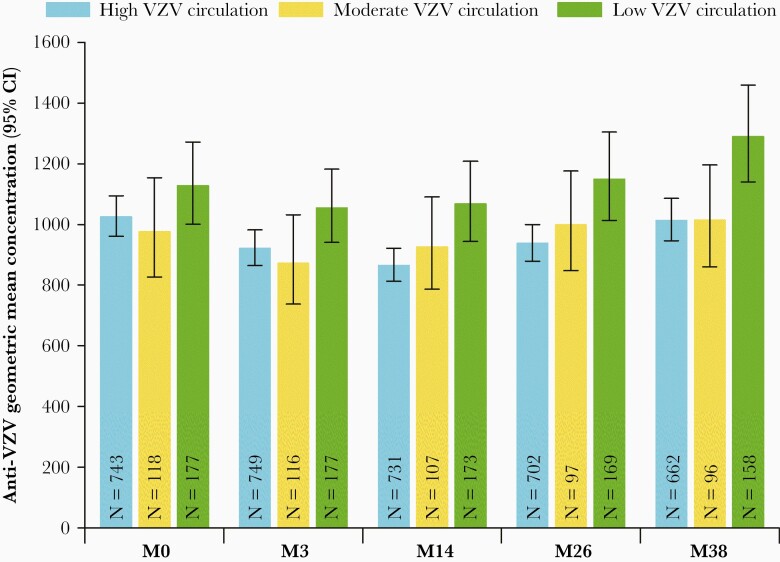
VZV-induced humoral immune responses by countries clustered according to VZV circulation (adapted per-protocol cohort for humoral immunity). Abbreviations: CI, confidence interval; M, month; N, number of participants; VZV, varicella-zoster virus.

With 1 exception (a participant from France at month 38), participants were seropositive for VZV antibodies at all time points. Anti-VZV GMCs for each country and time point are included in Supplementary Figure 1. A significant country effect on antibody titers was detected (*P* < .0001) and while antibody titers also varied between time points (*P* = .01), there was no significant interaction between time point and country, suggesting that the effect of time was clinically negligible. Therefore, further analyses were performed on data aggregated from all time points for each participant. Overall anti-VZV GMCs for each country are shown in Figure 2 and anti-VZV GMCs of countries clustered according to their presumed VZV circulation in Figure 3. The effect of VZV circulation was not significant after accounting for country heterogeneity.

Across all time points, overall GMCs were 950.13 mIU/mL (95% CI, 844.64–1068.79 mIU/mL), 968.04 mIU/mL (95% CI, 725.42–1291.80 mIU/mL), and 1127.99 mIU/mL (95% CI, 891.69–1426.91 mIU/mL) for countries with high, moderate, and low VZV circulation, respectively. While point estimates provided with the model for countries with high and moderate circulation were slightly lower compared to low circulation countries, the difference was not statistically significant.

### Anti-Varicella-Zoster Virus Cell-Mediated Immunity CMI Status

**Figure 4.**
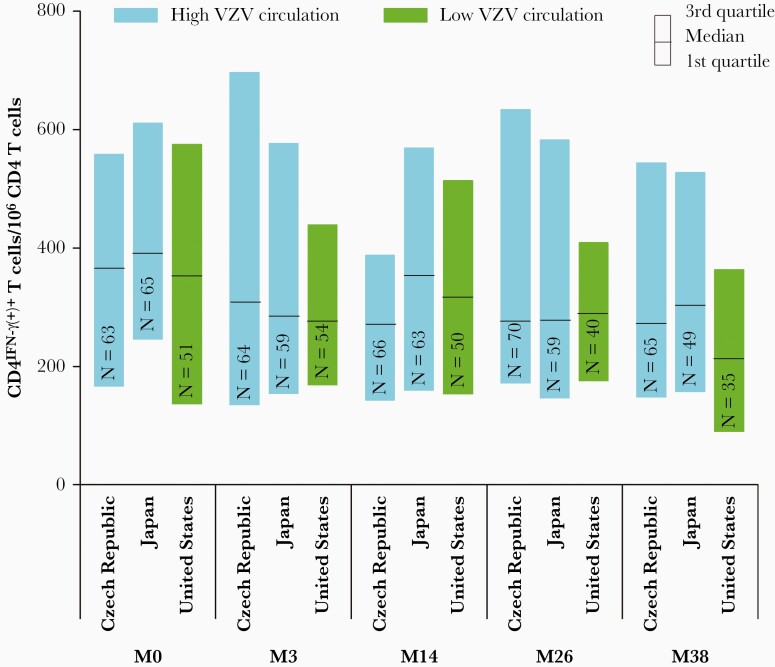
Descriptive statistic of the frequencies of VZV-specific CD4^IFN-γ+(+)^ T cells by country and time point (adapted per-protocol cohort for cell-mediated immunity). Abbreviations: IFN-γ, interferon-γ; M, month; N, number of participants with available results; VZV, varicella-zoster virus.

CMI samples were available from 3 countries: 2 with high and 1 with low circulation. CMI status at month 0, assessed by the mean frequency of CD4^IFN-γ+(+)^ T cells, was similar in placebo and vaccine recipients enrolled in ZOE-50 (463.38 [SD 426.07] versus 464.79 [SD 565.55]). A summary of CMI data is shown in Figure 4. CMI responses were analyzed descriptively at each time point for each country separately and these summaries did not suggest different distributions between time points. The country or VZV circulation level effect on the frequencies of VZV-specific CD4^IFN-γ+(+)^ T cells was not statistically significant (*P* = .56). Point estimates of the median frequency of VZV-specific CD4^IFN-γ+(+)^ T cells/10^6^ CD4 ^IFN-γ+(+)^ T cells counted were higher in the 2 countries with high circulation compared to the country with low circulation: 414 (95% CI, 334–495) for Czech Republic and 448 (95% CI, 364–532) for Japan versus 380 (95% CI, 289–472) for the United States.

### Cell-Mediated Immunity Increase Analysis

**Figure 5.**
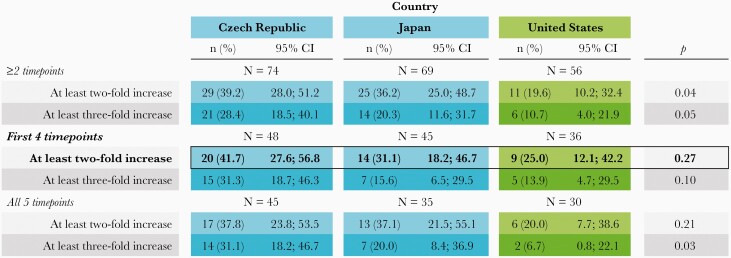
Cell-Mediated Immunity Increase Analysis (Bold) and Sensitivity Analyses in Participants With Results Available for ≥2, First 4, and All 5 Time Points (Adapted Per-Protocol Cohort for Cell-Mediated Immunogenicity). Blue box/dark gray box (print version), high varicella-zoster virus circulation; Green box/light gray box (print version), low varicella-zoster virus circulation. *P* values were obtained using a 2-sided Fisher exact test; as the cell-mediated immunity increase analysis was descriptive, *P* values can be regarded as inconclusive. Abbreviations: CI, confidence interval; n (%), number (%) of patients in a given category; N, number of participants with available results.

In the main analysis, based on participants with at least 2-fold increases and data available for the first 4 time points, no statistically significant difference was observed between countries with high and low VZV circulation (Figure 5). When sensitivity analyses were performed on the model for IFN-γ increases, the mean proportion of participants with CMI increases ranged from 7% to 42%; post hoc 2-sided *P* values below 5% were observed for the results of participants with data available for at least 2 time points (both for at least 2-fold and 3-fold increases) and all 5 time points (only for at least 3-fold increase). However, CIs were large and overlapping for all analyses.

## DISCUSSION

Concerns that universal varicella vaccination could result in increased herpes zoster incidence are based on the assumption that in varicella-endemic settings, large portions of the latently VZV-infected population experience periodic VZV exposure of sufficient intensity to stimulate the still unrecognized components of immunity that control reactivation [3]. However, this assumption remains untested and, even if it were validated, the magnitude of its effect at a population level may be modest [3]. To our knowledge, this is the first study to address this question by comparing humoral and cellular immunity as markers of VZV exposure in countries with different levels of circulating VZV. Overall, we found no consistent evidence that varicella vaccination reduces opportunities for VZV exposure in a general population of adults aged ≥50 years.

Changes in humoral immunity of adults following reexposure to VZV have been explored in several studies. Two studies [22, 23] found increases in 64% and 50% of reexposed adults. Antibody titers increased early after reexposure and resolved within a few weeks [22]. In contrast, Ogunjimi et al [24] observed no significant differences in humoral immunity between intensely exposed and control groups. A more recent study also failed to show increases in VZV-specific antibody titers over 1 year, or to find a statistically significant difference between VZV-reexposed grandparents and controls [25]. Whether these inconsistencies are caused by the difficulty of capturing short-lived changes in humoral immunity, or the fact that exposure does not consistently increase antibody levels, is still unclear.

Age-related decreases of VZV-specific T-cell frequencies are associated with increased risks of herpes zoster, although no proven correlates of protection have been identified [21]. Circulating VZV-specific T cells are long lived and can be detected at levels of approximately 0.1% CD4 T cells decades after primary infection [21], and repeated exposure to VZV has been shown to elicit persistently high CMI responses [26]. In our study, no significant difference was found between the frequencies of CD4^IFN-γ+(+)^ T cells of participants from countries with high versus low VZV circulation levels. This finding supports the hypothesis that VZV exposures in the general population are insufficient to elicit detectable immune responses in a significant proportion of individuals. Similar results were reported by Ogunjimi et al [25], who found no significant changes in frequencies of VZV-specific CD4^IFN-γ+^ T cells of grandparents with known reexposure to VZV over 1 year, or compared to controls.

The kinetics of circulating VZV-specific CD4 T cells are somewhat similar during symptomatic reactivation and following reexposure to VZV, reaching a peak within the first 2 to 4 weeks and then declining by week 6 to modestly elevated and persistent levels [21, 23]. In adults reexposed to VZV, an increase was observed in approximately 70% of all individuals [22, 23], while this proportion was much lower in older-aged grandparents, of whom only 25% showed increased CMI [25]. The latter estimate is in line with our results showing CMI increases in 7%–42% of participants. The higher age of participants included in the 2 studies might explain these lower values.

We observed transient CMI increases in all 3 countries with available data and although percentages were lower in the United States (a country with low VZV circulation), the differences were not statistically significant in the main analysis. In 3 of the 5 sensitivity analyses, differences between the United States and the 2 countries with high circulation (the Czech Republic and Japan) were marginally significant, although CIs were wide and overlapping for all analyses. While this could reflect reduced VZV exposure, it can also represent a country effect, considering that only 3 countries were included in this analysis. Although it is not clear whether endogenous boosting protects against development of herpes zoster [27, 28], it does increase parameters of humoral or cellular immunity [21] and could offer a potential explanation for the variability seen in our study. Overall, our results indicate that with the possible exception noted above, varicella vaccination does not have a significant effect on VZV immune status of people aged ≥50 years. It is also possible that the size of the long-term CD4 T-cell memory pool does not depend on increases, as it seems to revert to a specific set-point after reexposure, which would imply that these transient increases could be irrelevant for long-term protection.

In certain settings and subpopulations, the protective effects of reexposure to varicella seem quite possible. For example, in adults, a protective effect of living with children was observed in the prevaccination era but disappeared in the years after vaccination implementation [29], and a modest but long-lasting protective effect in household contacts of children with varicella was also found [30]. However, real-world evidence from the United States, a country with a well-established varicella vaccination program, does not seem to support these findings on a population-wide scale [3]. Despite the substantial drop in varicella incidence, no notable increase in herpes zoster rates have been observed in the general population, besides the steadily increasing trend that begun years before varicella vaccination [3]. In addition, a meta-analysis that assessed the population-level impact of varicella vaccination on herpes zoster incidence revealed that the number of hospitalized herpes zoster cases increased significantly only in the 10–49 years age group, but the effect was less than 2 additional cases per 100 000 persons [31]. These findings suggest that even a marked decline of VZV exposure produces more modest effects at a population level than those predicted by the Brisson model [6]. Our results are consistent with the possibility that—whether or not intense varicella exposures can, at times, protect against herpes zoster—at the population level, prior to introduction of varicella vaccination, opportunities for such exposures seem uncommon.

Our study has limitations. Humoral immune responses following VZV reexposure decline rapidly, with cellular immune responses being somewhat more durable [22]; the outcomes we evaluated may have therefore been insensitive markers of exposure. By performing analyses at several time points, we increased our odds for observing responses before they waned. However, the total 38-month observation time may have been too short to provide adequate opportunities for varicella exposure. All performed analyses were exploratory and no adjustment for multiplicity was possible. We only had CMI results for 2 countries with high VZV circulation and 1 with low circulation, allowing limited opportunities for comparison. As this was a post hoc exploratory analysis, sample size selection was not possible, and due to the modest number of individuals with CMI values available, some of our study outcomes lacked precision. Clustering of countries was performed based on the existence of universal varicella vaccination programs, which are proven to reduce varicella incidence, and not on actual reports of varicella incidence from each country. Finally, the effect of sociodemographic heterogeneity between the countries included in the analysis was not explored. However, CMI increase analysis was performed against a country-specific baseline, which might partially mitigate the issue.

In conclusion, we assessed humoral and CMI to determine whether VZV exposures are more common in adults aged ≥50 years in countries with widely circulating varicella versus countries with universal vaccination and good varicella control but found no consistent differences. Our results suggest a credible explanation why countries that have introduced varicella vaccination programs have not experienced postulated increases in herpes zoster incidence and can inform national authorities considering implementation of universal varicella vaccination. Repurposed immunological data from completed clinical trials can provide useful information with which to address important questions relating to the dynamics of infectious diseases.

## Supplementary Data

Supplementary materials are available at *The Journal of Infectious Diseases* online. Supplementary materials consist of data provided by the author that are published to benefit the reader. The posted materials are not copyedited. The contents of all supplementary data are the sole responsibility of the authors. Questions or messages regarding errors should be addressed to the author.

jiab500_suppl_Supplementary_Data

jiab500_suppl_Supplementary_Figure

## Notes

***Acknowledgments.*** The authors thank the Modis platform for editorial assistance and manuscript coordination, on behalf of GSK. Timea Kiss provided medical writing support and Julie Mellery coordinated the manuscript development and editorial support.

***Author contributions.*** S. C., G. C., R. v. d. M., and R. H. were involved in the post hoc conception and design. S. C., M. P., and B. C. performed the post hoc analyses. All authors had full access to post hoc and study data, contributed to data analysis or interpretation, and take responsibility for integrity and accuracy of data analysis. All authors reviewed the draft critically, approved the final submitted version, take accountability for all aspects of the published work, and the corresponding author had final responsibility to submit for publication. Drafts were developed by a professional publication writer according to the recommendations, documentation, and outline provided by authors.

***Financial support.*** This work was supported by GlaxoSmithKline Biologicals SA, which was involved in all stages of the study conduct and analysis and covered the costs associated with developing and publishing the present article.

***Potential conflicts of interest.*** S. C., B. C., M. P., and G. C. are employees of the GSK group of companies. S. C., B. C., and G. C. hold shares in the GSK group of companies as part of their employee remuneration. A. D. was employed by and held shares as part of his employee remuneration in the GSK group of companies during the conduct of this post hoc analysis and is a current employee of Bill and Melinda Gates Medical Research Institute. R. v. d. M. was employed by and held shares as part of his employee remuneration in the GSK group of companies during the conduct of this post hoc analysis and is a current employee of Coalition for Epidemic Preparedness Innovations. R. H. reports no potential conflicts.

Data sharing: The protocol of this study is available at www.gsk-studyregister.com (ID, 110390). GSK makes available the anonymized individual participant data and associated documents from interventional clinical studies which evaluate medicines upon approval of proposals submitted to www.clinicalstudydatarequest.com. To access data for other types of GSK sponsored research, for study documents without patient-level data, and for clinical studies not listed, please submit an enquiry via the website.

Presented in part: 35th Annual Meeting of the European Society for Paediatric Infectious Diseases, 23–27 May 2017, Madrid, Spain.
