# Supplementary material for: No Consistent Evidence of Decreased Exposure to Varicella-Zoster Virus Among Older Adults in Countries with Universal Varicella Vaccination
Source: J Infect Dis. 2021 Oct 5;225(3):413–21. doi: 10.1093/infdis/jiab500 (PMC8807177; doi:10.1093/infdis/jiab500)
Supplement: jiab500_suppl_Supplementary_Data [file jiab500_suppl_supplementary_data.docx]

**Supplementary Data**

**Inclusion criteria in the ZOE-50 study**

- Men or women aged 50 years or older at the time of the first vaccination;
- Provided written informed consent
- The investigator believed they would comply with the requirements of the protocol (e.g., completion of the diary cards/questionnaires, return for follow-up visits, have regular contact to allow evaluation during the study)
- Women could be enrolled in the study if:
- They were of non-childbearing potential, defined as current tubal ligation, hysterectomy, ovariectomy or post-menopause; OR
- They were of childbearing potential if they practiced adequate contraception for 30 days before vaccination, had a negative urine pregnancy test on the day of vaccination and agreed to continue adequate contraception during the entire treatment period and for two months after completion of the vaccination series

**Exclusion criteria in the ZOE-50 study**

- A history of herpes zoster
- A previous vaccination against varicella or herpes zoster, either with a registered product or from participation in a previous vaccine study (this included previous vaccination with childhood varicella vaccine)
- Any confirmed or suspected immunosuppressive or immunodeficient condition resulting from disease (e.g. malignancy, human immunodeficiency virus infection) or immunosuppressive/cytotoxic therapy (e.g. medications used during cancer chemotherapy, organ transplantation, or to treat autoimmune disorders)
- A history of allergic disease or reactions likely to be exacerbated by any component of the vaccine, which may include allergic reactions to other material or equipment related to study participation (e.g. materials that may possibly contain latex: gloves, syringes, etc.; however, the vaccine and vials in this study did not contain latex)
- Significant underlying illness that, in the opinion of the investigator, would be expected to prevent completion of the study (e.g., life-threatening disease likely to limit survival to less than four years)
- Concurrent participation in another clinical study, at any time during the study period, in which the participant had been or would be exposed to an investigational or a non-investigational product (pharmaceutical product or device)
- Used investigational or non-registered product (drug or vaccine) other than the study vaccine within 30 days preceding the first dose of study vaccine, or planned to use such a product during the study period
- Received immunoglobulins or any blood products within the 90 days preceding the first dose of study vaccine or planned to receive such products during the study period
- Administration or planned administration of any other immunizations within 30 days before the first or second study vaccination or scheduled within 30 days after study vaccination. However, licensed non-replicating vaccines (i.e., inactivated and subunit vaccines, including inactivated and subunit influenza vaccines for seasonal or pandemic flu, with or without adjuvant) could be administered up to 8 days before each dose or at least 14 days after any dose of study vaccine
- Any other condition (e.g., extensive psoriasis, chronic pain syndrome, cognitive impairment, severe hearing loss) that, in the opinion of the investigator, might interfere with the evaluations required by the study
- Acute disease or fever at the time of enrolment:
- Fever was defined as an oral, axillary, or tympanic temperature ≥37·5°C or a rectal temperature 38·0°C. The preferred route for recording temperature in this study was oral.
- Individuals with a minor illness (such as mild diarrhea, mild upper respiratory infection) without fever could be enrolled at the discretion of the investigator
- Chronic administration (defined as >15 consecutive days) of immunosuppressants or other immune-modifying drugs within six months prior to the first vaccine dose. For corticosteroids, prednisone <20 mg/day, or equivalent, was allowed. Inhaled and topical steroids were allowed
- Women were also excluded if they were:
- Pregnant or lactating, OR
- Planning to become pregnant or planning to discontinue contraceptive precautions (if of childbearing potential)
