# Supplementary material for: No Consistent Evidence of Decreased Exposure to Varicella-Zoster Virus Among Older Adults in Countries with Universal Varicella Vaccination
Source: J Infect Dis. 2021 Oct 5;225(3):413–21. doi: 10.1093/infdis/jiab500 (PMC8807177; doi:10.1093/infdis/jiab500)
Supplement: jiab500_suppl_Supplementary_Figure [file jiab500_suppl_supplementary_figure.docx]

**Supplementary Figure 1. VZV-induced humoral responses at each timepoint by country (Adapted per-protocol cohort for humoral immunity)**

**
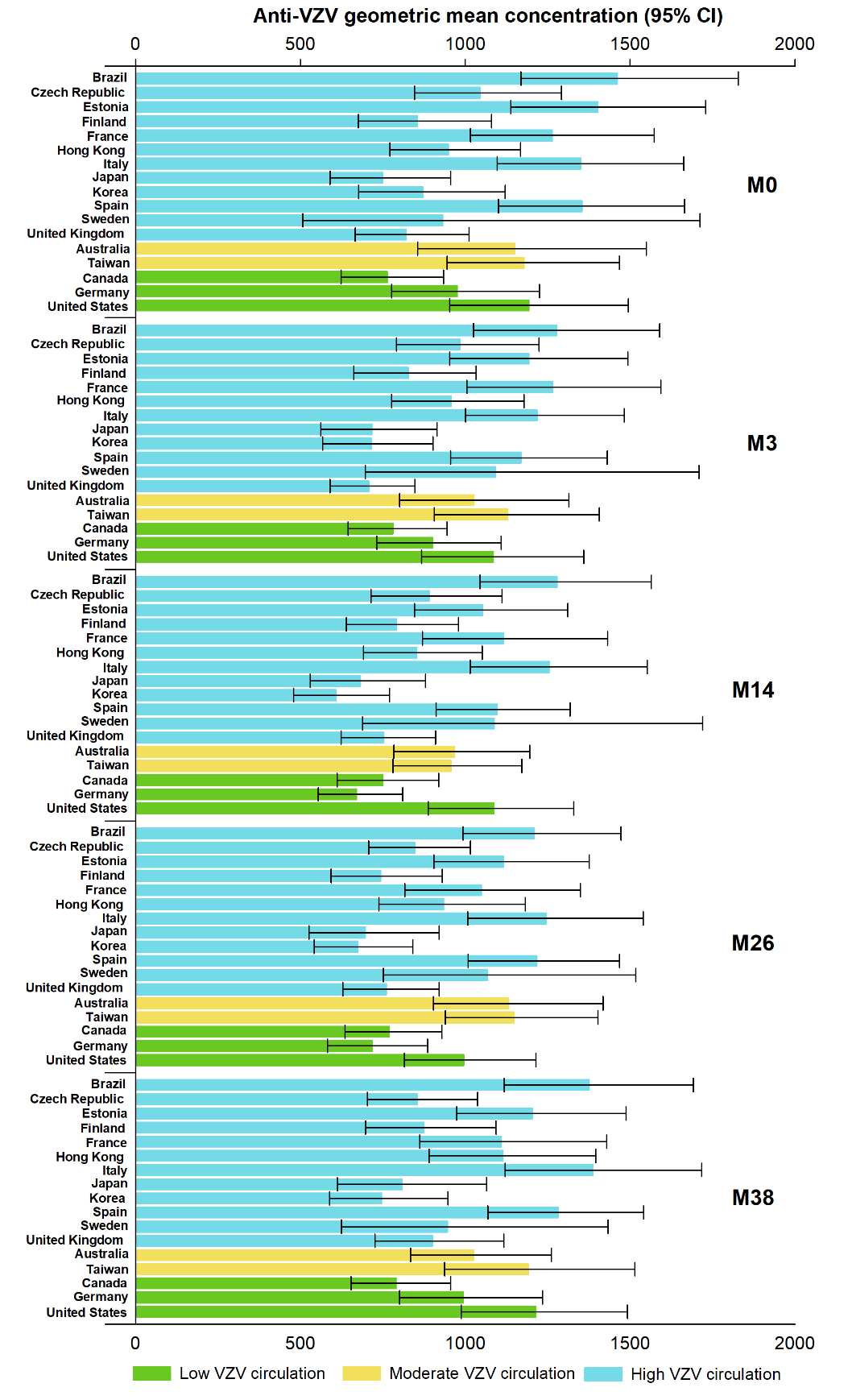
**

VZV, varicella-zoster virus; CI, confidence interval; M, month.
